# In-vivo characterization of macro- and microstructural injury of the subventricular zone in relapsing-remitting and progressive multiple sclerosis

**DOI:** 10.3389/fnins.2023.1112199

**Published:** 2023-04-11

**Authors:** Maria Cellerino, Simona Schiavi, Caterina Lapucci, Elvira Sbragia, Giacomo Boffa, Claudia Rolla-Bigliani, Serena Tonelli, Daniele Boccia, Nicolò Bruschi, Francesco Tazza, Diego Franciotta, Matilde Inglese

**Affiliations:** ^1^Department of Neurosciences, Rehabilitation, Ophthalmology, Genetics, Maternal and Child Health, University of Genoa, Genoa, Italy; ^2^Laboratory of Experimental Neurosciences, IRCCS Ospedale Policlinico San Martino, Genoa, Italy; ^3^Department of Neuroradiology, IRCCS Ospedale Policlinico San Martino, Genoa, Italy; ^4^Neuroimmunology Research Unit, IRCCS Mondino Foundation, Pavia, Italy; ^5^Department of Neurology, IRCCS Ospedale Policlinico San Martino, Genoa, Italy

**Keywords:** multiple sclerosis, subventricular zone, microstructural damage, neurogenesis, neuroprotection, remyelination

## Abstract

**Introduction:**

The subventricular zone (SVZ) represents one of the main adult brain neurogenesis niche. In-vivo imaging of SVZ is very challenging and little is known about MRI correlates of SVZ macro- and micro-structural injury in multiple sclerosis (MS) patients.

**Methods:**

The aim of the present study is to evaluate differences in terms of volume and microstructural changes [as assessed with the novel Spherical Mean Technique (SMT) model, evaluating: Neurite Signal fraction (INTRA); Extra-neurite transverse (EXTRATRANS) and mean diffusivity (EXTRAMD)] in SVZ between relapsing-remitting (RR) or progressive (P) MS patients and healthy controls (HC). We are also going to explore whether SVZ microstructural injury correlate with caudate (a nucleus that is in the vicinity of the SVZ) or thalamus (another well-defined grey matter area which is further from SVZ than caudate) volume and clinical disability. Clinical and brain MRI data were prospectively acquired from 20 HC, 101 RRMS, and 50 PMS patients. Structural and diffusion metrics inside the global SVZ, normal appearing (NA-) SVZ, caudate and thalamus were collected.

**Results:**

We found a statistically significant difference between groups in terms of NA-SVZ EXTRAMD (PMS>RRMS>HC; *p* = 0.002), EXTRATRANS (PMS>RRMS>HC; p<0.0001), and INTRA (HC>RRMS>PMS; *p* = 0.009). Multivariable models showed that NA-SVZ metrics significantly predicted caudate (*R*^2^ = 0.21, *p* < 0.0001), but not thalamus, atrophy. A statistically significant correlation between EXTRAMD and EXTRATRANS of the NA-SVZ and EDSS (*r*=0.25, *p*=0.003 and *r*=0.24, *p* = 0.003, respectively) was found. These findings were confirmed in analyses restricted to RRMS, but not to PMS patients.

**Discussion:**

In conclusion, the microstructural damage we observed within the NA-SVZ of MS patients – reflecting higher free water content (higher EXTRAMD), cytoarchitecture disruption and astrogliosis (higher EXTRATRANS and lower INTRA) - was more evident in the progressive as compared to the relapsing phases of MS. These abnormalities were significantly associated with a more pronounced caudate atrophy and higher clinical disability scores. Our findings may support the neuroprotective role of SVZ in MS patients.

## Background

Multiple Sclerosis (MS) is a chronic autoimmune disease characterized by the interaction of inflammatory, demyelinating, and degenerative processes within the CNS (Thompson et al., 2018b). Relapsing–remitting MS (RRMS) is the most common form of MS and is clinically characterized by the occurrence of clinical relapses followed by remitting phases of a neurological deficit, while progressive MS (PMS) is characterized by gradual accumulation of neurological disability independent of relapses (Thompson et al., 2018b). While inflammation has been recognized as the main ongoing pathological process during RRMS, disease progression seems to be primarily driven by neurodegeneration (Correale et al., 2016). A deeper understanding of the physio-pathological mechanisms leading to the failure of structural and functional recovery during disease progression remains of paramount importance in MS research. The variability of tissue repair is known to depend on several factors, including patients’ age, disease duration, lesion location and axonal integrity (Patrikios et al., 2006). Substantial remyelination is frequently observed during the earlier phases of MS and in younger individuals, whereas it is more sparse or even absent in PMS (Goldschmidt et al., 2009). In this context, the role of neural stem cells’ neurogenic and neuroprotective functions and of their possible damage occurring throughout disease course remain to be elucidated.

The subventricular zone (SVZ), immediately beneath the ependymal layer on the lateral wall of the lateral ventricles, contains multipotent neural stem cells. Together with the subgranular zone in the hippocampal dentate gyrus, the SVZ represents one of the main adult brain neurogenesis niche (Nait-Oumesmar et al., 2007; Wang et al., 2016) Although data regarding SVZ functioning mostly derive from animal studies, data in humans suggest that the SVZ persists in the adult human brain (Nait-Oumesmar et al., 2007), and SVZ astrocytes have been shown to retain the functions of neural stem cells *in vitro* (Nait-Oumesmar et al., 2008; Sanai et al., 2011). However, SVZ cellular organization is known to differ from rodents’ (Nait-Oumesmar et al., 2008; Sanai et al., 2011) and to change during human brain development, gradually reducing its neurogenic functions in the passage from infant to adolescent and adult age (Sanai et al., 2011; Coletti et al., 2018). Although the capacity of SVZ cells to proliferate and migrate *in vivo* is still a matter of debate (Sanai et al., 2011), recent findings suggest that in normal conditions and after injury the adult human SVZ can generate mature astrocytes (David-Bercholz et al., 2021). Importantly, neurogenesis in humans has been shown to rapidly decline with increased age, supposedly due to a loss of function of adult stem cell and progenitor cell populations (Segel et al., 2019; Butt et al., 2022) in line with that, elderly individuals are more susceptible to neurodegenerative disease such as Parkinson’s (PD) and Alzheimer’s (AD) diseases (Wang et al., 2016). On the other hand, there is growing evidence that neurodegenerative diseases may affect SVZ cell proliferation (Curtis et al., 2007). Furthermore, it has been suggested that SVZ-derived neural precursor cells may contribute to repair and recovery from ischemic stroke (Williamson et al., 2019). Interestingly, post-mortem studies have identified mature and immature oligodendrocytes and oligodendrocyte precursor cells (OPCs) in some chronic MS lesions, suggesting a mobilization of SVZ-derived progenitors mainly to peri-ventricular lesions (Nait-Oumesmar et al., 2008). SVZ-derived cells have also been shown to contribute to remyelination through various mechanisms in murine models of MS (Etxeberria et al., 2010; Samanta et al., 2015; Liu et al., 2016). At the same time, accumulating evidence showed that the SVZ might also exert non neurogenic functions via the secretion of soluble growth factors, the release of neuroprotective molecules and the modulation of microglia functions (Mosher et al., 2012; Butti et al., 2014).

*In-vivo* imaging of SVZ is very challenging and very little is known about MRI correlates of SVZ macro- and micro-structural injury in multiple sclerosis (MS) patients. In the past years, several magnetic resonance imaging (MRI) techniques have been proposed to characterize microstructural alterations due to tissue disruptions caused by MS as well as to study the CNS capability to respond to intrinsic and extrinsic injury by reorganizing its structure and function. In fact, diffusion MRI (dMRI) has been shown to be able to detect the anisotropic movement of water molecules inside the voxel and to be sensitive to axonal density and dispersion, cell shape as well as the presence of potential inflammation. Thanks to dMRI, microstructural changes in myelin and neuroaxonal integrity in the cortex and white matter have been shown to occur even in the early stages of MS and to correlate with neurological disability (Granberg et al., 2017). Our group recently confirmed the role of the dMRI metrics in the characterization of lesions and normal appearing white matter (NAWM) tissue in different stages of the disease, showing a globally more severe degree of tissue damage in progressive as compared to relapsing MS patients (Schiavi et al., 2021). Among all the proposed multicompartment diffusion models, the Spherical Mean Technique (SMT) is a novel approach able to estimate microscopic features specific to the intra- and extra-neurite compartment of the CNS, not confounded by fiber crossing or orientation dispersion (Kaden et al., 2016a) particular, for each voxel it provides information on neurites signal fraction, axonal directions and their diffusivities, as well as extra-axonal diffusivities (Kaden et al., 2016a). For this reason, SMT has been successfully applied to characterize brain (Bagnato et al., 2020) and spinal cord (By et al., 2018) pathological changes in MS patients.

The objective of our study was to obtain an in-vivo characterization of SVZ in MS patients with advanced MRI techniques. Since the presence of MS lesions in the periventricular areas - hence possibly involving the SVZ - is a hallmark of MS, and considering that the widespread tissue damage worsens in the progressive phases of the disease, we predicted that microstructural alterations involving SVZ are present in MS patients and increase with the progression of the disease. We accordingly expected to observe a microstructural disruption in the SVZ – radiologically similar to that occurring in other MS lesions and in the NAWM - in MS patients, more evident in the progressive with respect to the relapsing–remitting stages of the disease. Assuming that this hypothesis held true, we sought to explore whether the microstructural injury found could impact the neuroprotective functions of SVZ. To that end - as exploratory analyses – we investigated its correlations with caudate/thalamus nuclei volume (considering the SVZ position, we expected to find a stronger association with caudate rather than with thalamus volumes, since the first is closer to the SVZ, and we thus expected it could more easily benefit from its neuroprotective functions) and clinical disability. More specifically, the aims of our study were to:

(I) explore differences in terms of volume and microstructural characteristics in SVZ between RRMS patients, PMS patients and healthy controls (HC);
(II) investigate whether SVZ microstructural characteristics may help predict the volume of caudate nucleus - that is in the vicinity of the SVZ - and of thalamus - as representative of another well-defined grey matter area which is further from SVZ than caudate nucleus; we chose to conduct this analysis in order to exclude that the possible association between the damage of the SVZ and that of caudate nucleus was non-specific and driven by a global NAWM damage and/or a diffuse brain atrophy;
(III) investigate whether SVZ microstructural characteristics’ correlate with patients’ disability status.

## Methods

### Population

A total of 151 patients (101 RRMS, 50 PMS) and 20 sex- and age-matched HC were prospectively enrolled. The PMS population included both primary (n = 32) and secondary (n = 18) PMS patients. Inclusion criteria for patients were (I) age 18–70 years (II) MS diagnosis according to the 2017 revisions to McDonald’s criteria (Thompson et al., 2018a) and (III) Expanded Disability Status Scale (EDSS; Kurtzke, 1983) score ≤ 7. For HC, only the criterion (I) was required. Exclusion criteria for all subjects were (I) other neurological comorbidities and (II) contraindications to MRI.

All patients underwent neurological examination with the assessment of the EDSS score. On the same day, all subjects underwent brain MRI. The study was approved by the local ethical committees and written informed consent was obtained from all participants according to the Declaration of Helsinki.

### MRI acquisition

All patients underwent MRI on a Siemens Prisma 3 T scanner (Erlangen, Germany) with a 64-channel head and neck coil. The MRI protocol included: (i) 3D sagittal T2-FLAIR (176 slices, repetition time/inversion time/echo time (TR/TI/TE): 5,000/1,800/393 ms; resolution 0.4 × 0.4 × 1 mm^3^); (ii) 3D sagittal T1 MPRAGE (208 slices, TR/TI/TE: 2,300/919/2.96 ms; resolution 1 × 1 × 1 mm^3^;); (iii) twice-refocused spin echo echo-planar imaging sequence for multi-shell diffusion-weighted images (80 slices, TR/TE: 4,500/75 ms; distributed in 5 shells with b-value = 300, 700, 1,000, 2,000, 3,000 s/mm^2^ in 3, 7, 16, 29, 46 directions each, plus 7 b-value = 0 images acquired with both anterior–posterior and posterior–anterior phase encoding directions; spatial resolution 1.8 × 1.8 × 1.8 mm^3^).

### MRI analysis

#### Lesions and SVZ segmentation

FLAIR and T1 lesions were manually segmented using Jim software (Jim 7.0, Xinapse System; http://www.xinapse.com), creating FLAIR and T1 lesion masks, respectively.

**Figure 1:**
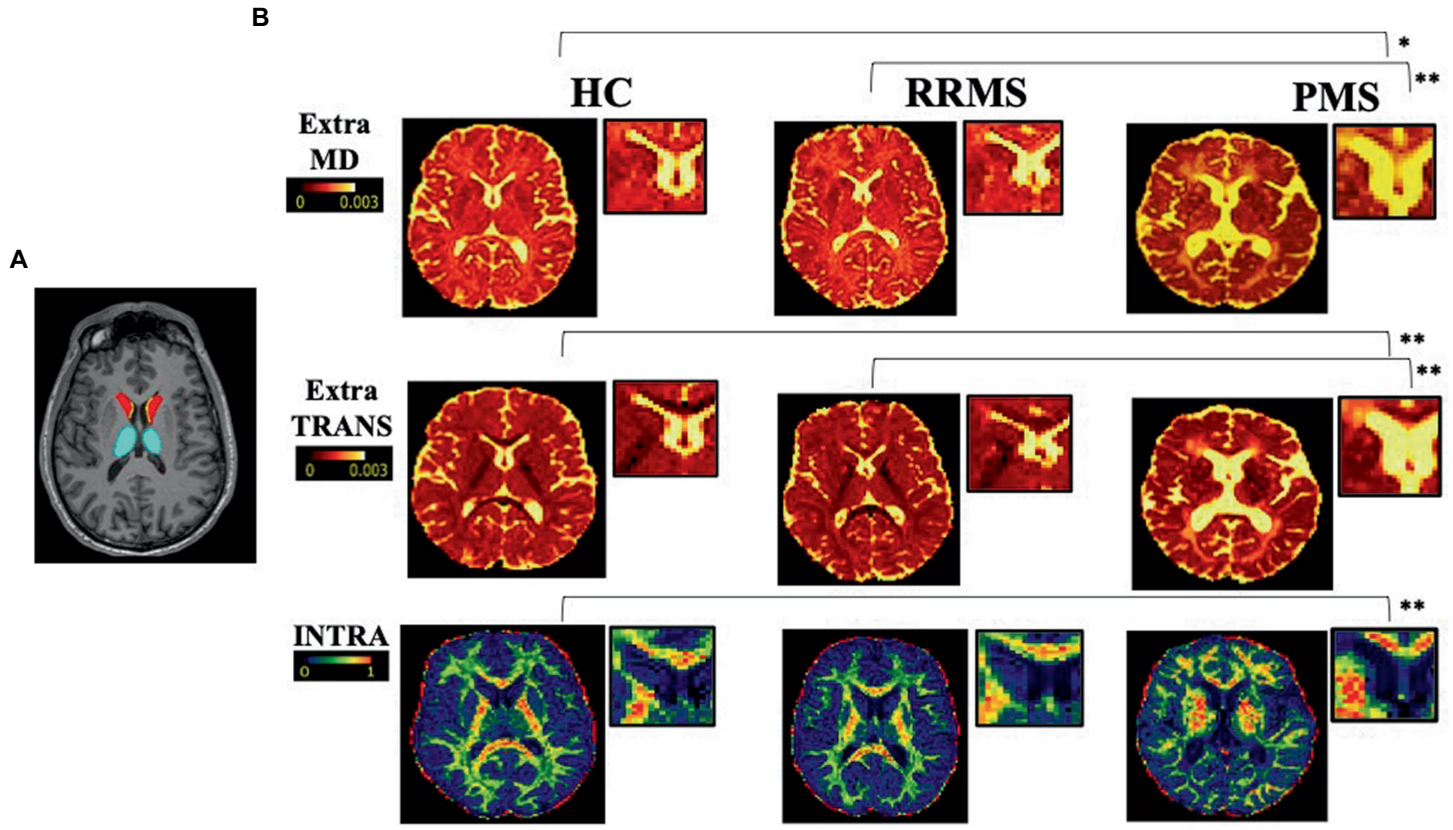
Selected examples of axial slice in a healthy control, a RRMS and a PMS patient. On the left **(A)** a selected axial slices from T1-weighted image showing segmented masks of SVZ (yellow), caudate (red), and thalamus (light blue). On the right **(B)** representative axial slices of EXTRAMD (top; 10–3 mm^2^/ms), EXTRATRANS (center; 10–3 mm^2^/ms), and INTRA (bottom) maps in a HC, a RRMS and a PRS patient. *; **p-values referring to the analyses performed on the global population (*p < 0.5 and **p < 0.01). HC = healthy control; RRMS = relapsing–remitting multiple sclerosis; PMS = progressive multiple sclerosis;

Given the anatomical information provided in Cherubini et al. (2010) we segmented a mask of SVZ on high-resolution T1-weighted images in a standard space (Montreal Neurological Institute space) and then non-linearly co-registered on native T1- and diffusion-weighted images through FSL non-linear registration tool [FNIRT (Jenkinson et al., 2012); Figure 1]. We then obtained the normal appearing SVZ tissue (NA-SVZ) by masking out the volume occupied by FLAIR lesions.

Thalamus and Caudate subcortical structures were segmented using FIRST (Patenaude et al., 2011). Finally, to avoid any overlap between the masks, all segmentations were then checked on native T1 of each subject.

Total intracranial volume (TIV) and grey matter (GM) volume were obtained using CAT12, part of SPM.^1^

All segmentations were manually performed by a neurologist with experience in neuroimaging of MS (CL) and checked by another rater (GB).

#### Diffusion processing

Diffusion MR images were first denoised using the Marchenko-Pastur principal component analysis algorithm (Veraart et al., 2016) available in MRtrix3 (Tournier et al., 2019). Then they were corrected for movement artifacts and susceptibility induced distortions using eddy and top-up commands from FMRIB Software Library (FSL; Andersson et al., 2003; Smith et al., 2004; Andersson and Sotiropoulos, 2016). As last step of pre-processing we also performed B1 field inhomogeneity correction to all the dMRI volumes (Tustison et al., 2010). To compute the microstructural maps derived from SMT model, we used the open-source code available at https://github.com/ekaden/smt. To improve the registration of structural masks in diffusion space, once the SMT maps were obtained we up-sampled them from original resolution of 1.8 × 1.8 × 1.8 mm^3^ to the higher T1 resolution (1x1x1 mm^3^); we simply regrid the SMT maps to match T1 resolution without interpolation to reduce registration errors. Then, diffusion weighted images were rigidly registered on T1- weighted images using FLIRT with boundary-based registration (Greve and Fischl, 2009), and the resulting transformations were inverted and applied to the masks to register them in the diffusion weighted image space. All the registrations were visually checked and eventually edited by a trained professional with more than 5 years of experience in neuroimaging to avoid mislabeling of clear CSF voxels. Finally, we extracted the mean values inside global SVZ, SVZ-NA, NAWM, caudate and thalamus of the following SMT metrics: the microstructural maps of neurite signal fraction (INTRA), extra-neurite transverse diffusivity (EXTRATRANS), and extra-neurite mean diffusivity (EXTRAMD) that describe the intracellular compartment, the anisotropic extra-neurite compartment including its transverse microscopic diffusivity and the mean diffusion outside the axons, respectively (Kaden et al., 2016a,b).

### Statistics

Descriptive results were reported as mean with standard deviation (SD) or median with interquartile range (IQR). Demographic differences between groups were analyzed using Chi-square, Mann–Whitney and independent samples t-test where appropriate. Differences in terms of MRI metrics between RRMS, PMS patients and HCs were assessed by analysis covariance (ANCOVA) adjusting for age and gender; for volumes comparison, total intracranial volume (TIV) was added to the covariates; for the comparison of each SMT metric within NA-SVZ between groups, analyses were also adjusted for the average microstructural damage in the NAWM. Bonferroni corrected post-hoc analyses (to adjust for multiple comparisons) were performed. Multivariable linear regression models adjusted for age and gender explored associations between normal appearing SVZ microstructural metrics and the ratio of caudate and thalamus volumes with total GM volume in the global MS population and in RRMS or PMS patients, separately, as well as in the HC group; analyses regarding patients were additionally adjusted for FLAIR lesion volume. In line with the literature (Chard et al., 2002), we chose to explore the association of NA-SVZ microstructural injury with caudate/GM and thalamus/GM ratio (calculated as the ratio between caudate and thalamus nuclei volume - respectively – and total GM volume) instead of the volume of the single nuclei to account for the relationship between total GM and the volumes of caudate and thalamus. Partial Spearman correlation models explored the association between SVZ microstructural characteristics and EDSS, adjusting for age and gender. All *p*-values were two-sided and considered statistically significant when *p* ⩽ 0.05. *p*-values after Bonferroni correction are provided for the comparisons of MRI metrics between groups. Given their exploratory nature, other analyses were not adjust for multiple comparisons. SPSS 22 (IBM; version 22.0) was used for computation.

## Results

### Population characteristics

Demographic and clinical data are reported in Table 1. A total of 151 patients (101 RRMS and 50 PMS; 99 female; mean age 43 ± 11 years, mean disease duration 43 ± 9 years; median EDDS 2.5) and 20 HC (11 female; mean age 42 ± 13.6 years) were included. Although the global MS population and HC were matched in terms of age and gender, as expected gender composition and age differed between the three groups, as the PMS population had a higher percentage of male patients and older age as compared to other groups; these variables were consequently included as a covariate in all the analyses comparing groups. As expected, progressive patients presented higher EDSS scores than relapsing subjects (*p* < 0.0001). Disease duration was similar between the two groups of patients.

**Table 1:** Demographics and clinical characteristics.

	MS *N* = 151	RRMS *N* = 101	PMS *N* = 50	HC *N* = 20		
Demographics					*p*-values*	*p*-values**
Age, mean (SD), y	43 (11.6)	39 (10.5)	51 (8.6)	42 (13.6)	<0.0001	HC vs. RRMS: 0.748 HC vs. PMS: 0.001 RRMS vs. PMS: <0.001
Female, no (%)	99 (66%)	74 (73%)	25 (50%)	11 (55%)	0.01	HC vs. RRMS: 0.103 HC vs. PMS: 0.705 RRMS vs. PMS: 0.005
Clinical characteristics					*p*-values***	
Disease duration, mean (SD), y	11 (8.8)	10 (8.3)	12 (9.7)	-	0.2	-
EDSS score at baseline, median (range)	2.5 (0–7)	2 (0–6.5)	4.5 (1.5–7)	-	<0.0001	-

RRMS, relapsing–remitting multiple sclerosis; PMS, progressive multiple sclerosis; HC, healthy controls; SD, standard deviation; y, years; EDSS, expanded disability status scale.

* *p*-values for the RRMS vs. PMS vs. HC comparison; ANOVA (age), Chi-square (gender).

** *p*-values for the single groups comparison; Bonferroni corrected post-hoc analyses of ANOVA (age), Chi-square (gender).

*** *p*-values for the RRMS vs. PMS comparison; Mann–Whitney (disease duration, EDSS).

### MRI characteristics in HC, RRMS, and PMS patients

Mean values and differences (Bonferroni corrected p-values) between groups in terms of MRI characteristics are reported in Table 2.

**Table 2:** Volumes and microstructural characteristics of SVZ, caudate and thalamus nuclei in HC, RRMS, and PMS patients.

Volumes		
	Volume (ml), mean (SD)	*p*-value^a^
Caudate		
HC	4.90 (0.79)	**HC vs. RRMS 0.009** **HC vs. PMS 0.013** RRMS vs. PMS 0.539
RRMS	4.37 (0.78)	
PMS	4.09 (0.83)	
Thalamus
HC	10.98 (12.28)	**HC vs. RRMS 0.001** **HC vs. PMS <0.0001** RRMS vs. PMS 0.999
RRMS	9.34 (17.59)	
PMS	8.96 (14.40)	
Global SVZ		
HC	1.34 (0.25)	HC vs. RRMS 0.999 HC vs. PMS 0.999 RRMS vs. PMS 0.240
RRMS	1.25 (0.25)	
PMS	1.29 (0.26)	
NA-SVZ		
HC	1.34 (0.25)	HC vs. RRMS 0.553 HC vs. PMS 0.999 RRMS vs. PMS 0.245
RRMS	1.22 (0.26)	
PMS	1.26 (0.29)	
FLAIR lesion volume		
HC	n.a.	RRMS vs. PMS 0.707
RRMS	10.75 (10.85)	
PMS	12.49 (11.55)	
SMT metrics
	EXTRAMD (10^−3^mm^2^/s)	EXTRATRANS (10^−3^mm^2^/s)	INTRA
Caudate			
HC	1.41 (0.0001)	1.15 (0.0001)	0.38 (0.0456)
RRMS	1.42 (0.0002)	1.18 (0.0002)	0.36 (0.0555)
PMS	1.56 (0.0002)	1.22 (0.0003)	0.34 (0.0660)
*p*-value^b^	HC vs. RRMS 0.999 HC vs. PMS 0.115 RRMS vs. PMS 0.108	HC vs. RRMS 0.999 HC vs. PMS 0.065 RRMS vs. PMS 0.123	HC vs. RRMS 0.306 HC vs. PMS 0.064 RRMS vs. PMS 0.766
Thalamus			
HC	1.38 (0.0001)	1.09 (0.0001)	0.42 (0.0292)
RRMS	1.42 (0.0001)	1.17 (0.0002)	0.38 (0.0539)
PMS	1.50 (0.0001)	1.25 (0.0002)	0.37 (0.0537)
*p*-value^b^	HC vs. RRMS 0.557 **HC vs. PMS 0.014** RRMS vs. PMS 0.077	HC vs. RRMS 0.103 **HC vs. PMS 0.001** RRMS vs. PMS 0.082	**HC vs. RRMS 0.002** **HC vs. PMS <0.0001** RRMS vs. PMS 0.928
NAWM			
HC	1.16 (0.0222)	0.85 (0.0290)	0.46 (0.0217)
RRMS	1.17 (0.0272)	0.88 (0.0472)	0.47 (0.0338)
PMS	1.16 (0.0271)	0.85 (0.0562)	0.44 (0.046)
*p*-value^b^	HC vs. RRMS 0.290 HC vs. PMS 0.999 RRMS vs. PMS 0.333	HC vs. RRMS 0.083 HC vs. PMS 0.999 RRMS vs. PMS 0.057	HC vs. RRMS 0.134 HC vs. PMS 1.000 **RRMS vs. PMS 0.032**
NA-SVZ			
HC	1.32 (0.0001)	1.03 (0.0001)	0.43 (0.0471)
RRMS	1.35 (0.0002)	1.09 (0.0002)	0.39 (0.0638)
PMS	1.50 (0.0022)	1.12 (0.0002)	0.38 (0.0649)
*p*-value^b^	HC vs. RRMS 0.999 **HC vs. PMS 0.017** **RRMS vs. PMS 0.003**	HC vs. RRMS 0.999 **HC vs. PMS 0.005** **RRMS vs. PMS 0.001**	HC vs. RRMS 0.360 **HC vs. PMS 0.008** RRMS vs. PMS 0.101

RRMS, relapsing–remitting multiple sclerosis; PMS, progressive multiple sclerosis; HC, healthy controls; SD, standard deviation; SVZ, sub-ventricular zone; SVZ-NA, normal appearing sub-ventricular zone; NAWM, normal appearing white matter.

a Bonferroni corrected p-values for the post-hoc analyses of ANCOVA adjusted for age, gender and total intracranial volume (TIV).

b *p*-value for the Bonferroni corrected post-hoc analyses of ANCOVA adjusted for age and gender; for each SMT metrics within NA-SVZ, analyses were also adjusted for the average microstructural damage in the NAWM. Statistically significant *p*-values are reported in bold.

While no differences in global SVZ and SVZ-NA volumes between HC, RRMS and PMS emerged at ANCOVA analyses (*p* = 0.188 and *p* = 0.145, respectively), as expected caudate and thalamus nuclei volumes differed between HC, RRMS and PMS patients (*p* = 0.016 and *p* < 0.0001, respectively). Post-hoc analysis showed statistically significantly smaller caudate and thalamus volumes in PMS (*p* = 0.013 and *p* < 0.0001, respectively) and smaller thalamus volume in RRMS (*p* = 0.001) patients with respect to HC. FLAIR lesion volumes were, as expected, higher in PMS than in RRMS patients; however, such difference did not reach statistical significance.

As per microstructural characteristics, we observed that EXTRAMD, EXTRATRANS and INTRA metrics within SVZ-NA were different among HC, RRMS and PMS patients (*p* = 0.002, *p* < 0.0001 and *p* = 0.009 respectively). At post-hoc analysis, EXTRAMD was higher in PMS with respect to RRMS and HC (*p* = 0.017 and *p* = 0.003 respectively), EXTRATRANS was higher in PMS with respect to RRMS and HC (*p* = 0.005 and *p* = 0.001 respectively), INTRA was lower in PMS with respect to HC (*p* = 0.008). When considering the total NAWM, EXTRATRANS and INTRA significantly differed between groups (*p* = 0.017 and *p* = 0.015, respectively), while EXTRAMD did not (*p* = 0.124); at post-hoc analysis only a statistically significant difference between RRMS and PMS patients in terms of INTRA (*p* = 0.032) emerged.

In the caudate, EXTRATRANS was the only SMT metric which differed between HC, RRMS and PMS patients (*p* = 0.042); no differences in EXTRAMD and INTRA metric were found (*p* = 0.055 and *p* = 0.071 respectively); no statistically significant differences emerged at post-hoc analysis. All SMT metrics in the thalamus significantly differed between groups (EXTRAMD *p* = 0.012; EXTRATRANS *p* = 0.002; INTRA *p* < 0.0001). Similarly to SVZ-NA, post-hoc analyses showed higher EXTRAMD and EXTRATRANS and lower INTRA in PMS patients as compared to HC (*p* = 0.014, *p* = 0.001 and *p* < 0.0001, respectively); INTRA. was lower in PMS also with respect to RRMS (*p* = 0.002).

### Correlation of SVZ microstructural characteristics with caudate and thalamus nuclei volume

Given the difference observed in terms of SMT metrics between HC and different groups of patients, we sought to investigate whether such microstructural properties were associated with the volumes of caudate and thalamus nuclei (expressed as fractions between caudate/thalamus and total GM volume). Results of the analyses exploring this association are shown in Table 3.

**Table 3:** Multivariate analyses exploring the association between NA-SVZ microstructural properties and caudate and thalamus GM ratio.

	Caudate/GM ratio		Thalamus/GM ratio	
HC (*n* = 20)
	B (95% CI)	*p* value	B (95% CI)	*p* value
EXTRAMD	−51838.31 (−134827.50–31150.88)	0.202	−108840.13 (−230249.9–12569.65)	0.075
EXTRATRANS	58918.21 (−32080.55–149916.98)	0.187	119826.76 (−13300.71–252954.35)	0.074
INTRA	55.82 (−24–26 – 135.91)	0.157	120.25 (3.09–237.42)	0.054
Total Cohort (*n* = 151)
	B (95% CI)	*p* value	B (95% CI)	*p* value
EXTRAMD	−15164.21 (−31409.17 – −1080.74)	0.067	−1834.49 (−29149.14–25840.15)	0.895
EXTRATRANS	16276.21 (−1315.43–33867.84)	0.070	371.84 (−29207.14–25480.83)	0.980
INTRA	17.23 (1.27–33.19)	0.035	5.84 (−20.00–32.68)	0.667
RRMS (*n* = 101)
	B (95% CI)	*p* value	B (95% CI)	*p* value
EXTRAMD	−21022.17 (−39363.11 – −2681.22)	**0.025**	4415.65 (−24877.48–33708.78)	0.765
EXTRATRANS	22578.48 (2512.08–42644.87)	**0.028**	−7426.52 (−39475.44–24622.39)	0.647
INTRA	22.41 (4.41–40.41)	**0.015**	−1.36 (−30–11 – 27.38)	0.925
PMS (*n* = 50)
	HR (95% CI)	*p* value	B (95% CI)	*p* value
EXTRAMD	3092.43 (−35574.22–41759.08)	0.873	19275.51 (−50362.36–76313.38)	0.682
EXTRATRANS	−2448.31 (−43604.95–38780.32)	0.905	−13475.68 (−80892.27–53940.91)	0.689
INTRA	2.94 (−34.51–40.40)	0.875	−1.87 (−62.23–59.49)	0.951

*n* = number; B = non standardized coefficient; 95% CI = confidence intervals; GM = grey matter; RRMS = relapsing–remitting multiple sclerosis; PMS = progressive multiple sclerosis; NA-SVZ = normal appearing sub-ventricular zone. Statistically significant *p*-values are reported in bold.

Considering the global population, the multivariable model exploring NA-SVZ metrics as predictors of caudate/GM volumes’ ratio was able to explain approximately 20% of the variance in the outcome (R^2^ 0.21, *p* < 0.0001), being INTRA the only microstructural metric of the SVZ independently contributing to the model (*p* = 0.035). When analyses were restricted to RRMS patients the model remained statistically significant (R^2^ 0.3, *p* < 0.0001), and we found that all the SMT metrics were significantly associated with the outcome (EXTRAMD *p* = 0.025, EXTRATRANS *p* = 0.028, INTRA *p* = 0.015). When analyses were restricted to PMS patients, the model was not statistically significant (*p* = 0.448).

Applying the same multivariate models, none of the SMT metrics of the NA-SVZ appeared to be significantly associated with thalamus/GM ratio in the global population, nor when analyzing RRMS and PMS patients separately.

The same analyses performed in the HC population found no associations between SMT metrics of NA-SVZ and caudate/GM (*p* value of the model: 0.126) or thalamus/GM (*p* = 0.100) ratio volumes.

### Correlations between SVZ microstructural characteristics and patients’ disability status

**Figure 2:**
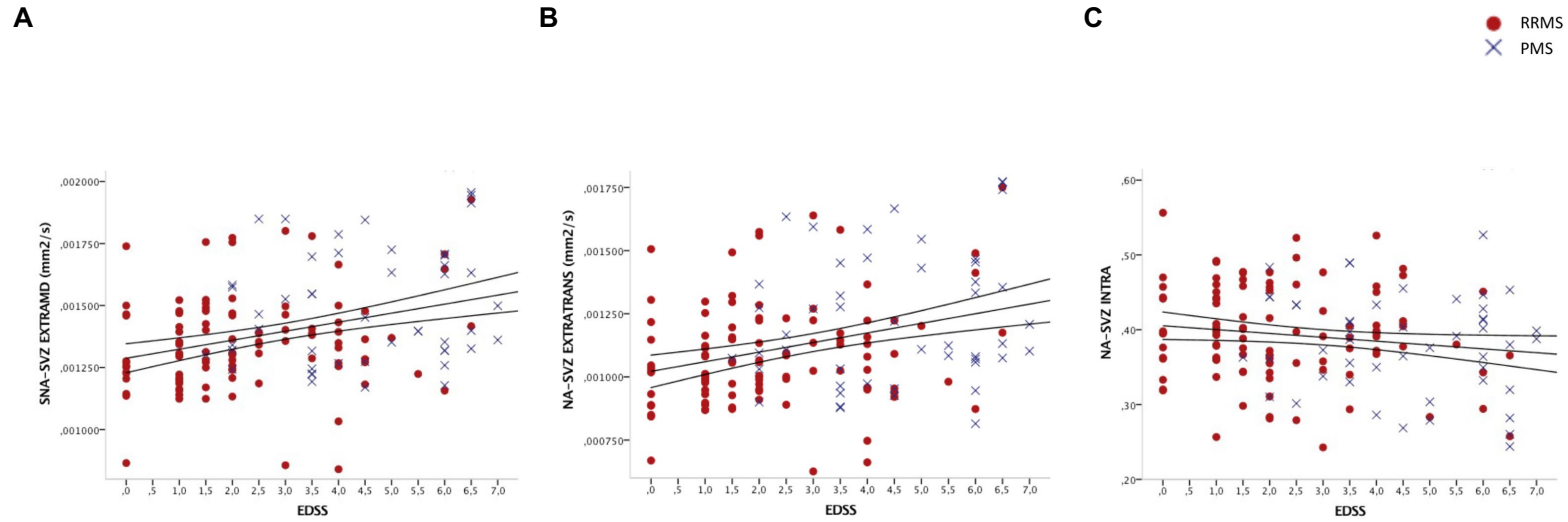
Scatter plot showing associations between **(A)** EXTRATRAMD of the NA-SVZ and EDSS; **(B)** EXTRATRANS of the NA-SVZ and EDSS; and **(C)** INTRA of the NA-SVZ and EDSS. EDSS = expanded disability status scale; RRMS = relapsing–remitting multiple sclerosis; PMS = progressive multiple sclerosis; NA-SVZ = normal appearing sub-ventricular zone Axes represent residuals. Linear fit and 95% confidence intervals are shown.

Considering the global population, we observed a statistically significant correlation between EXTRAMD and EXTRATRANS of the NA-SVZ and EDSS (*r* = 0.25, *p* = 0.003 and *r* = 0.24, *p* = 0.003, respectively). The correlation between NA-SVZ EXTRAMD/EXTRATRANS and EDSS remained significant even when considering only RRMS patients (*r* = 0.20, *p* = 0.044 and *r* = 0.21, *p* = 0.039, respectively), but none of the microstructural characteristics of the SVZ significantly correlated with EDSS in PMS patients. A graphical representation of these correlations is reported in Figure 2.

## Discussion

Chronic demyelination and axonal loss are recognized phenomena driving disease progression in MS (Thompson et al., 2018b). In animal models, SVZ is recognized as an area that might contribute to CNS regeneration following tissue damage, exerting both neurogenic and neuroprotective functions through the proliferation, differentiation and maturation of neural cell precursors among which OPCs, the release of soluble molecules (neurotrophic cytokines/growth factors) that act on the survival of local oligodendrocytes, and by modulating microglia (Mosher et al., 2012; Butti et al., 2014). In humans, SVZ functions are known to decline during brain development and aging. Nevertheless, accumulating evidence suggest that adult human SVZ might represent a site of major modifications in response to neurological diseases, (Butt et al., 2022) thanks to SVZ-derived precursor cells recruitment into remyelinating oligodendrocytes and SVZ-mediated secretion of soluble neuroprotective molecules. It suggests that SVZ impairment might have a role in neurodegenerative processes (Sim et al., 2002). However, most data derive from animal studies or in-vitro characterization of SVZ. Indeed, in-vivo characterization of SVZ is very challenging, possibly due to the limited resolution of MRI images which prevents the possibility of isolating the small cellular layers such as those that compose the SVZ (Cherubini et al., 2010). Against this background, in a recent paper Cherubini et al. were able to radiologically characterize the SVZ microstructural damage in patients with dementia, and more specifically reported a progressive increase of mean diffusivity, reflecting a defect in neurogenesis, in the SVZ of patients with mild cognitive impairment and AD (Cherubini et al., 2010).

Given the paucity of information available regarding MRI correlates of SVZ in MS, the main objective of the present study was to obtain an *in vivo* characterization of macro- (i.e., volume) and microstructural (i.e., SMT metrics) injury of the SVZ in MS patients and to explore the clinical implications of such damage. The hypotheses that (I) a microstructural disruption in the SVZ characterizes MS patients, (II) that these alterations are even more evident in the progressive stages and (III) that such microstructural injury correlate with caudate nucleus volume and clinical disability are supported by the results of our study.

Initially, we found statistically significant differences in all SMT metrics when comparing HC, RRMS and PMS. Specifically, we observed a continuous increase (HC < RRMS<PMS) in EXTRAMD and EXTRATRANS, and a decrease (HC > RRMS>PMS) for INTRA. In the healthy brain, the SVZ consists of an ependymal layer, a gap region and an astrocytic ribbon, and it is separated from the caudate nucleus and the striatum by a layer of myelin; it contains three different cell types (types A, B and C) that are organized in a specific pattern, with the type A cells closest to the ependymal layer, the type B cells forming the astrocytic ribbon and the type C cells located close to the myelin layer and the striatum (Curtis et al., 2007). An interconversion of cell types has been shown to occur in different neurodegenerative diseases (Curtis et al., 2007), and an infiltration of T cells in the SVZ of aging human brains, both under non-pathological conditions and in neurodegenerative contexts (as it could be considered progressive MS) has been demonstrated (Moreno-Valladares et al., 2020). The variations in terms of SMT metrics that we observed during the different phases of MS might reflect similar pathological processes, being suggestive of higher free water content (higher EXTRAMD, indirectly reflecting ongoing inflammatory processes), cytoarchitecture disruption and astrogliosis (higher EXTRATRANS and lower INTRA, indirectly reflecting a higher degree of cellular damage and fiber disruption).

Although older age in PMS patients could be a possible confounding factor, since neurogenesis in humans is known to decline with increased age, it is worth noting that all our analyses were adjusted for age. Moreover, the two groups of patients did not differ in terms of disease duration. Thus, although still preliminary, our findings seem to indicate that microstructural SVZ involvement is related to disease stage. This is in line with recent results of Bodini et al. (2016), who found a borderline impact of age on dynamic remyelination as assessed with positron emission tomography (PET) and no significant effect for disease duration. Our results contribute to an ongoing debate regarding the relationship between neuro-repair potential, age and disease duration (Nsi et al., 2002; Patrikios et al., 2006; Bodini et al., 2016; Jäkel et al., 2019).

Interestingly, in the caudate and thalamus nuclei a similar microstructural damage together with reduced volumes (HC > RRMS>PMS) were both observed. However, similarly to Cherubini et al. (2010), we found no significant change in the volume of SVZ between the groups of patients. Although the mis-segmentation of the periventricular white matter could be one of the possible explanations (due to the previously mentioned limited resolution of MRI), considering our results we could also speculate that microstructural alterations reflecting tissue dysfunction within the SVZ might precede its atrophy.

The choice to use SMT to investigate the possible pathological substrate of SVZ injury in different phases of MS relied on its interesting basic assumptions and its encouraging recent results in MS (Kaden et al., 2016a; By et al., 2018; Lakhani et al., 2020). Overcoming the issue represented by the fixed intrinsic diffusivity of other multicompartment models (Lakhani et al., 2020), SMT considers WM as a two-compartment (intra- and extra-axonal) tissue and provides signal fraction and diffusion metrics per axon without confounds from fiber direction, crossing, or dispersion (Kaden et al., 2016a). This model provided validation against axonal histology in the tuberous sclerosis animal model, which is more suitable for detecting CNS axonal injury than the MS animal model because it is free from the inflammatory component. Importantly, SMT has been shown to reliably quantify axonal content without artifactual effects from fiber-crossing and orientation dispersion. Since such model is applicable to any condition affecting myelin and axonal integrity, SMT has been already applied *in vivo* in different studies focusing on the brain (Bagnato et al., 2020) and spinal cord (By et al., 2018) of MS patients, demonstrating to be helpful in differentiating MS lesions damage from the NAWM as well as the NAWM of MS patients from that of healthy subjects (Lakhani et al., 2020) and in characterizing pathological features within MS lesions (Devan et al., 2020). Overall, our findings showed a certain degree of tissue disruption characterizing the SVZ region of MS patients, and a more evident damage in the progressive as compared to the relapsing phases of MS.

Once we characterized the microstructural alterations of SVZ, we sought to investigate whether they were associated with lower volumes of other deep nuclei. Indeed - in normal conditions - SVZ heterogeneous cells are maintained in an environment that is permissive to gliogenesis with neuroprotective potentialities, and responds to neurodegenerative insults in adjacent brain regions by increasing progenitor cell proliferation in an attempt to limit the process of neurodegeneration (Curtis et al., 2007). The capacity of SVZ progenitor cells to proliferate, migrate and exert their neurogenic effect in favor of the caudate nucleus has been demonstrated in only animal models of Huntington’s disease (Curtis et al., 2007). In humans, and particularly in an MS context, the SVZ response to nearby occurring insults is potentially restricted to gliogenesis and production of neurotrophic factors. We thus speculated that the microstructural damage that we observed in MS patients could impact SVZ protective functions. Considering the SVZ position, we expected to find a stronger association with caudate rather than with thalamus volumes, since the first is closer to the SVZ. Our findings confirmed the hypothesis and pointed at INTRA as the SMT metric with the strongest association with caudate volume. Indeed, lower INTRA values within the NA-SVZ - indirectly reflecting an abnormal cytoarchitecture and astrogliosis – was significantly associated with lower caudate volumes. These findings were confirmed in analyses restricted to RRMS but not to PMS patients, suggesting that a more severe microstructural damage (as we observed in PMS vs. RRMS patients) might account for a loss of SVZ capability to enhance neuroprotection.

In parallel to its neuroprotective functions, SVZ in supposed to have a role in remyelination (Franklin and Ffrench-Constant, 2017; Jäkel et al., 2019). Accordingly, we speculated that the SVZ tissue damage could be one of the processes that affect remyelination efficiency in MS, especially in its progressive phases. Remyelination failure necessarily contribute to the progressive disability that characterizes the later stage of MS. Accordingly, we sought to investigate the correlation between SVZ microstructural characteristics and global disability. We decided to use EDSS as it represents the most widely used measure to assess clinical disability, thus indirectly reflecting the capacity of patients to recover from relapses. In a recently published paper (Bodini et al., 2016), Pittsburgh compound B ([11C]PiB) PET was shown to allow quantification of myelin dynamics in MS and to enable stratification of patients depending on their individual remyelination potential. Importantly, authors showed that the index of dynamic remyelination was strongly associated with EDSS scores in relapsing MS patients, suggesting that an efficient remyelination process is one of the discriminating factors in determining a better prognosis in MS. In line with this hypothesis, we observed a significant correlation between SVZ microstructural properties and patients’ disability status (higher EXTRAMD and EXTRATRANS - indirectly reflecting more inflammation and cell disruption - positively correlated with EDSS scores), which was maintained in RRMS but not in PMS patients. Our findings are in line with the observations of Rasmussen et al. (2011), who showed that in a relapsing model of experimental autoimmune encephalomyelitis (EAE), the neural stem cells (NCS) became activated and initiated regeneration during the acute disease phase, but lost this ability during the chronic phase (Rasmussen et al., 2011). Various mechanisms have been proposed to enhance NSCs proliferation and mobilization, and consequently promote tissue repair (Samanta et al., 2015; Remaud et al., 2017; Sulehria et al., 2020) in murine model of MS. However, when it comes to patients, enhancing neuroprotection and neurorepair remains an important, but still elusive, therapeutic goal.

The main limitation of our study is that, given the cross-sectional nature of the study, we cannot exclude the SVZ alterations are a single epiphenomenon of the diffuse damage of the NAWM and that they do not actually reflect a greater/lesser ability to remyelinate. However, the microstructural alterations we found in the area of SVZ were stronger as compared to those observed in the global NAWM, suggesting that our results could be just partly explained by a non-specific global NAWM damage and/or a diffuse brain atrophy. Another important limitation represented by the risk of erroneous identification of SVZ due to its small size and location on MRI images, as an effect of partial volume. In fact, even if we up-sampled the diffusion weighted images to avoid mislabeling due to registration, the original resolution at which the maps were computed was 1.8 mm isotropic so some voxels might still be affected by partial volume. Other limitations of our study are the lack of spinal cord images, which could strongly affect the disability status, and the relatively small sample size. Lastly, the evidence for neurogenesis in the SVZ beyond infancy in humans remains limited, although some data suggesting the capacity of the adult human SVZ to generate mature astrocytes are reported in the literature (David-Bercholz et al., 2021). Our findings need to be confirmed by larger analyses, which should include longitudinal data. Assessing the correlation between SVZ measures and lesion characteristics (number, volume and distribution) would be also of added value to further elucidate the role of SVZ in MS patients.

Although there are limitations to our analyses, our data suggest that the SVZ injury as assessed with MRI is evident in all phases of MS and becomes even more evident with disease progression. Moreover, based on our exploratory and still preliminary findings, we might speculate that such microstructural injury reflects a loss of SVZ function resulting in compromised neuroprotection and neurorepair, possibly contributing to disease progression.

In conclusion, despite the reported limitations, we believe that our results may contribute to elucidate mechanisms underlying neuroprotection and tissue repair. The advent of new therapeutical strategies with the potential to promote remyelination requires the identification of patient-specific factors that may influence the response and the optimal therapeutic window for neurorepair. The advanced diffusion MRI modeling used in the present study might not only provide novel insight for understanding the pathophysiology of MS, but possibly also enable stratification and monitoring of patients based on their neurorepair potential for current and experimental treatments choice.

## Data availability statement

The raw data supporting the conclusions of this article will be made available by the authors, without undue reservation.

## Ethics statement

The studies involving human participants were reviewed and approved by CER LIGURIA: 74/2020 - DB ID 10346. The patients/participants provided their written informed consent to participate in this study.

## Author contributions

MC: acquisition of clinical data, analyzed the data, interpreted the data, and drafted the manuscript for intellectual content. SS: analyzed the MRI data and interpreted the data. CL: acquisition of clinical and MRI data and interpreted the data. ES, GB, DB, NB, and FT: acquisition of clinical and MRI data. CR and ST: acquisition of MRI data. DF: contributed to the design of the study and revised the manuscript for intellectual content. MI: designed and conceptualized study, interpreted the data, and revised the manuscript for intellectual content. All authors contributed to the article and approved the submitted version.

## Funding

This work was supported by grants from Italian Ministry of Health (Rete delle Neuroscienze e della Neuroriabilitazione).

## Conflict of interest

The authors declare that the research was conducted in the absence of any commercial or financial relationships that could be construed as a potential conflict of interest.

The reviewer GP declared a past co-authorship with the authors SS and MI to the handling editor.

## Publisher’s note

All claims expressed in this article are solely those of the authors and do not necessarily represent those of their affiliated organizations, or those of the publisher, the editors and the reviewers. Any product that may be evaluated in this article, or claim that may be made by its manufacturer, is not guaranteed or endorsed by the publisher.

## Footnotes

^1^ https://neuro-jena.github.io/cat/
